# The Efficacy of Pilates on Urinary Incontinence in Korean Women: A Metabolomics Approach

**DOI:** 10.3390/metabo11020118

**Published:** 2021-02-19

**Authors:** Gyumin Kang, Haelim Lee, Malsoon Shin, Jaekwan Kim, Sungki Lee, Youngja Park

**Affiliations:** 1 School of Bio-Medical Science, Korea University, 2511 Sejong-ro, Sejong 30019, Korea; kgm1905@gmail.com; 2 Department of Sports Medicine, College of Health Science, CHA University, 120 Haeryong-ro, Donggyo-dong, Pocheon 11160, Korea; HLim@cha.ac.kr; 3 School of Global Sport Studies, Korea University, 2511 Sejong-ro, Sejong 30019, Korea; malsoon@korea.ac.kr; 4 College of Pharmacy, Korea University, 2511 Sejong-ro, Sejong 30019, Korea; jkvision1101@naver.com; 5 Korea Basic Science Institute, Seoul 02841, Korea; 6 Omics Research Center, 2511 Sejong-ro, Sejong 30019, Korea

**Keywords:** pelvic floor muscle (PFM), Oov Pilates, core exercise, urinary symptom, xMWAS, untargeted metabolomics, liquid chromatography-mass spectrometry (LC-MS), amino acid, exercise biomarker, exercise metabolism

## Abstract

Pilates has been known as exercise intervention that improves the function of pelvic floor muscle (PFM) associated with impacting urinary incontinence (UI). This study investigated the effect of Pilates on UI in Korean women by determining the change in functional movement of PFM (FMP) and metabolic profiles. UI group with Pilates (UIP, *n* = 13) participated in 8-weeks Oov Pilates program, and 8 subjects were assigned to Control and UI group with no Pilates (UINP), respectively. Before and after 8 weeks, plasma samples were collected from all participants, and ultrasonography was used to measure the functional change of PFM for calculating FMP ratio. Plasma samples were analyzed by mass spectrometry to identify the change of metabolic features. After 8-weeks intervention, FMP ratio was remarkably decreased in UIP (48.1% ↓, *p* < 0.001), but not in Control and UINP (*p* > 0.05). In metabolic features, L-Glutamine (*m*/*z*: 147.07 [M + H]^+^), L-Cystathionine (*m*/*z*: 240.09 [M + NH_4_]^+^), L-Arginine (*m*/*z*: 197.1 [M + Na]^+^), and L-1-Pyrroline-3-hydroxy-5-carboxylate (*m*/*z*: 147.07 [M + NH_4_]^+^) were significantly elevated solely in UIP (*p* < 0.001). Our study elucidated that Pilates can ameliorate the FMP and enhance the specific metabolic characteristics, which was potentially associated with invigorated PFM contractility to effectively control the bladder base and continence.

## 1. Introduction

Urinary incontinence (UI) is a medical symptom known as an involuntary leakage of urine [1], and has shown a prevalence rate of between 4 and 8% in world population [2]. In 2018, the total number of population experiencing UI was estimated at 420 million, consisting of 300 million women and 120 million men. More importantly, epidemiological approaches also asserted that UI symptoms can prevalently occur in female population [3] primarily between 45 and 59 years old [4]. Occurrence of UI was associated with complex neuro-muscular mechanisms to control the bladder muscles and voiding functions [3], and can also be caused by the sarcopenia or progressive muscle atrophy in pelvic floor muscle (PFM) [5]. Considering that surgical interventions for UI were found to have a profound impact on the quality of life of middle-aged people, and may accompany medical complications after the invasive treatments [3], alternative non-invasive approaches using medications and medical devices were recommended to manage the UI symptoms [3].

Previous studies reported that exercise interventions such as Kegel and CrossFit program [6,7] could provide a beneficial leverage to ameliorate the dysfunctional muscle groups that induce UI symptoms. Based on the score of life quality, PFM training (PFMT) programs could play a pivotal role as a first-line intervention in women experiencing UI [8], which improves the strength, endurance, power, and relaxation in PFM group [9]. In this context, given that Pilates has been considered as a specialized physical activity to strengthen and improve the PFM function [10,11,12,13], clinical benefits of Pilates on UI need to be scientifically verified by means of novel scientific method.

Metabolomics is the comprehensive analytical study of chemical compounds within cells, bio-fluids, and tissues; interactions within a biological property and metabolism can be collectively explained by metabolites known as a final product of biological metabolism. In particular, untargeted metabolomics can create “metabolomic snapshot” that provides the unbiased identification of thousands of metabolites, whereas targeted metabolomics focuses on quantifying the pre-determined metabolites of interest [14] As metabolomics have developed into cornerstone of systems biology to scientifically depict the large-scaled analysis of metabolites [15], the field of metabolomics could be powerful experimental impetus to understand further the metabolic responses to physical activities such as Pilates. Studies also showed that technological advancements in metabolomics could provide the underlying mechanisms and changed metabolic phenotype not only in demanding exercise conditions [16,17] but also in clinical-settings, diabetes, and hypertension [18,19].

To date, studies showed that Pilates has positive kinesiological effects on isokinetic exercise performance, postural balance, chronic low back pain, and body composition of sedentary obese women [20,21,22,23] whose methodological approaches mainly concentrated on macroscopic advantages derived from Pilates. Metabolomics has a scientific capability to discover the phenotypic information of living organism among the various metabolic pathways [24], therefore changed metabolic characteristics in UI following Pilates program can be fundamentally understood, indicating that microscopic aspects of Pilates can be confirmed in terms of “Sportomics,” interdisciplinary field combined with omics and sports science [25]. As biological information and change regarding cellular metabolisms can be verified by the metabolomics [24,26,27], it is speculated that high resolution untargeted metabolomics will pin-point the effect of Pilates on UI through discovering the change of existing metabolic biomarkers.

Therefore, the primary aim of our study is to explicate the potential efficacy of Pilates on the functional change of PFM and what metabolic features can be changed in Korean women experiencing UI after 8-weeks Oov Pilate under the high resolution untargeted metabolomics technology using liquid chromatography-mass spectrometry (LC-MS) quadrupole time-of-flight (Q-TOF).

## 2. Results

### 2.1. Anthropometrical Data and PFM Function of Subjects

In body mass (kg), BMI (kg/m^2^), and body fat (%), there were no significant differences between pre and post-intervention in all groups. FMP ratio in UIP was significantly decreased (48.1% ↓) after 8-weeks Pilates (*p* < 0.05), but not in Control (7.4% ↓) and UINP (8.7% ↓) (*p* > 0.05) as shown in Table 1. No statistical differences was observed in the age (year) and height (cm) (*p* > 0.05) among the three groups (Table 1).

### 2.2. Discrimination of Metabolic Profiles in and between Pre- and Post-Intervention

**Figure 1:**
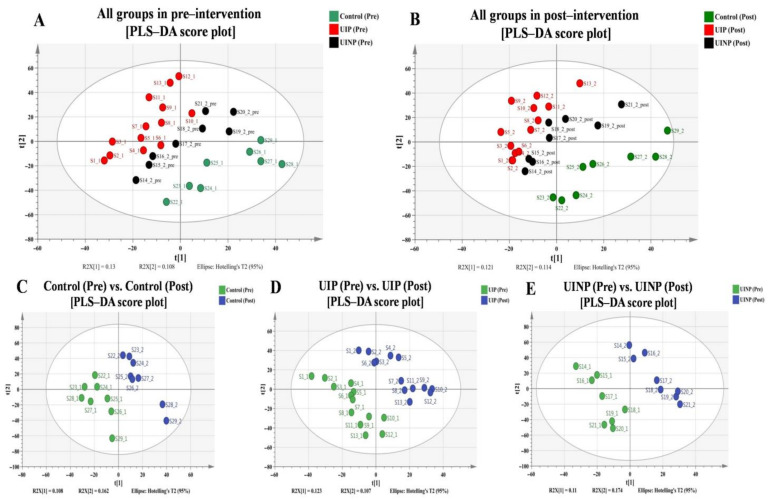
Multivariate statistical analysis using PLS-DA among all groups in pre-intervention, all groups in post-intervention, and between pre- and post-intervention in each group. PLS-DA: partial least squares discriminant analysis; Control: control group; UIP: urinary incontinence group with Pilates; UINP: urinary incontinence group with no Pilates; t [1]: first principal component score; t [2]: second principal component score; R2X: cumulative variance contribution rate; Hotelling’s T2: indication that all samples are located within 95% confidence interval.

To confirm the discrimination of metabolic profiles among all groups in pre-intervention (Figure 1A), all groups in post-intervention (Figure 1B), and between pre- and post-intervention in each group (Figure 1C–E), median-summarized 6574 metabolic features obtained from xMSanalyzer were analyzed by SIMCA 14.1 (Umetrics AB, Umeå, Sweden) with unit variance (UV) scaling function. When performing the discriminative analytical approach for all cases, the explanatory and predictive ability in each case of model were evaluated based on R^2^ and Q^2^, respectively, where the numerical value for perfect model is defined as 1.0 (100.0%) [28]. As shown in Figure 1C–E, discriminative analysis was individually conducted for each group; visualized configurations demonstrated a distinct separation between pre- and post-intervention, which indicates differentiated metabolic characteristics between two intervention time-points. To evaluate the validity of the model, R^2^ and Q^2^ in Control, UIP, and UINP were calculated: Control (R^2^: 99.9%, Q^2^: 83.3%), UIP (R^2^: 99.7%, Q^2^: 77.9%), and UINP (R^2^: 99.9%, Q^2^: 71.6%) (Figure 1C–E), indicating that all produced models are not over-fitting, and are reliable. However, in discriminative analysis of metabolic profiles among all groups allocated in two interventional time-points, marginal predictability was found in all models: pre- (R^2^: 94.2%, Q^2^: 26.3%) and post-intervention (R^2^: 93.4%, Q^2^: 12.7%) in all groups (Figure 1A,B).

### 2.3. Integrative and Differential Network Analysis between Metabolic Features and FMP Ratio

**Figure 2:**
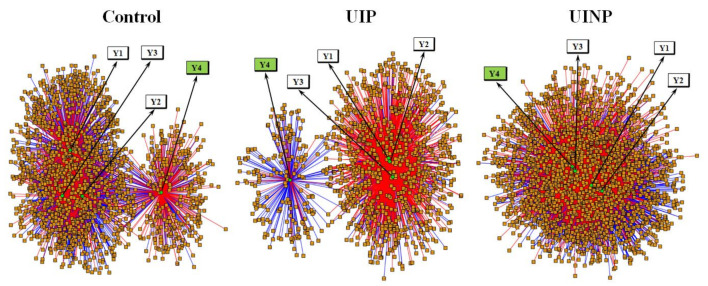
Integrative and differential network analysis between metabolic profiles and anthropometrical data/FMP ratio in Control, UIP, and UINP. Control: control group; UIP: urinary incontinence with Pilates; UINP: urinary incontinence with no Pilates; Y1: body mass; Y2: BMI; Y3: body fat percentage; Y4: FMP ratio; green circular node: anthropometrical data and FMP ratio (Y1, Y2, Y3, and Y4); orange squared node: metabolic profiles; blue edge: negative correlation between node variables; red edge: positive correlation between node variables.

Integrative and differential network analysis was performed using xMWAS (v0.552) that provides the community detection and visualized correlations (Blue and red edge) between measured metabolites (value of *m*/*z*) with metabolic intensity and comparative data (Body mass, BMI, body fat percentage, and FMP ratio). A total of 399, 214, and 647 metabolic features in control, UIP, and UINP, respectively, was positively and negatively correlated with FMP ratio (Y4) based on applying the correlational threshold of 0.5 and *p*-value threshold of Student’s *t*-test set at 0.05. Among the total metabolites, UIP had 180 metabolic features that showed a negative correlation with Y4, which accounts for the highest percentage (84.1%, 180 negatively correlated features in total) compared to Control (47.1%, 188 negatively correlated features in total) and UINP (39.5%, 256 negatively correlated features in total). As shown in Figure 2, when comparing to Control and UINP, UIP showed the most conspicuous independent community with dense negative correlation clusters between metabolic profiles and FMP ratio (Y4).

### 2.4. Identification of Metabolic Pathways Associated with FMP Ratio

**Figure 3:**
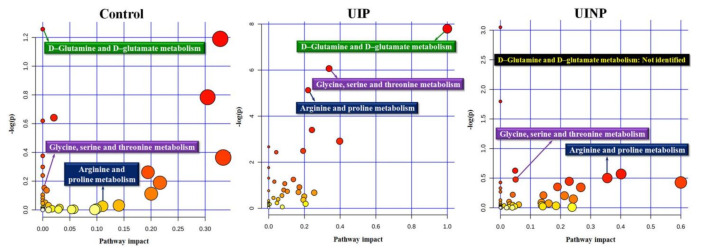
Identified metabolic pathways associated with FMP ratio in Control, UIP, and UINP. Control: Control group; UIP: Urinary incontinence group with Pilates; UINP: urinary incontinence group with no Pilates; pathway impact: statistical value calculated based on the metabolic importance of matched metabolites in each pathway, which uses the pathway topology analysis using the relative-betweeness centrality.

In all groups, to identify the metabolic pathways that can be found between metabolic profiles and FMP ratio (Y4), a total number of positively and negatively correlated metabolic profiles (Control: 399; UIP: 214; UINP: 647 features) with *m*/*z*, R/T, and ionic intensity were produced by xMWAS and consecutively entered into HMDB (https://hmdb.ca) (accessed on 15 November 2020) to acquire the identifier of human metabolome, “KEGG ID” obtained by annotating the metabolic features with *m*/*z* and R/T. In MetaboAnalyst 4.0, 404, 168, and 496 annotated metabolites for Control, UIP, and UINP, respectively, were used to produce the metabolic pathway and related statistical information. When the top three significant primary pathways in UIP were statistically confirmed (Table 2 and Figure 3), those were designated as a comparison criteria that can be compared with that of Control and UINP. As shown in Table 2, D-glutamine and D-glutamate metabolism in UIP had the highest pathway impact (PI) (PI = 1.0, *p* = 0.0004) compared to Control (PI = 0 and *p* = 0.2845) and UINP (Not identified). In UIP, glycine, serine and threonine (*p* = 0.0023) and arginine and proline metabolism (*p* = 0.0059) were also significantly identified in UIP, but not in both Control and UINP (*p* > 0.05).

### 2.5. Change in Identified Metabolic Features Associated with FMP Ratio

**Figure 4:**
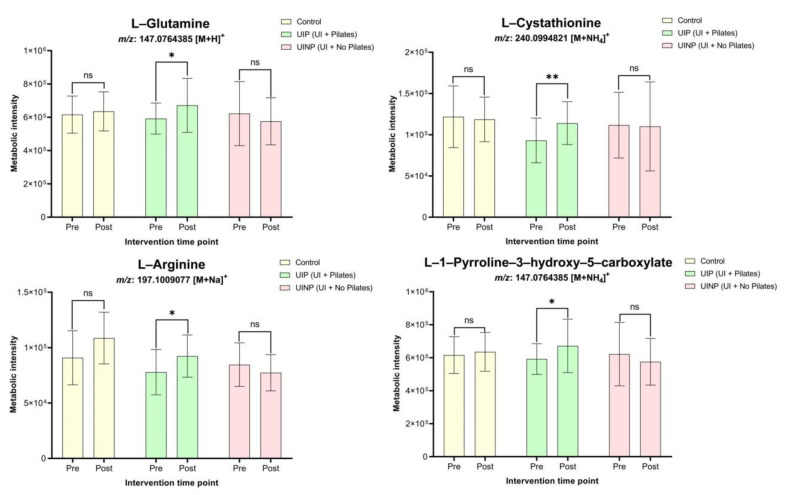
Change in identified metabolic features associated with FMP ratio between pre- and post-intervention in Control, UIP, and UINP. Control: control group; UIP: urinary incontinence group with Pilates; UINP: urinary incontinence group with no Pilates; *m*/*z*: mass-to-charge ratio in ions that quantifies molecules in chemical or biological mixture. Pre: before 8-weeks intervention; post: after 8-weeks intervention. * (*p* < 0.05): significantly different from Pre. ** (*p* < 0.001): significantly different from Pre. ns means non-significant.

**Figure 5:**
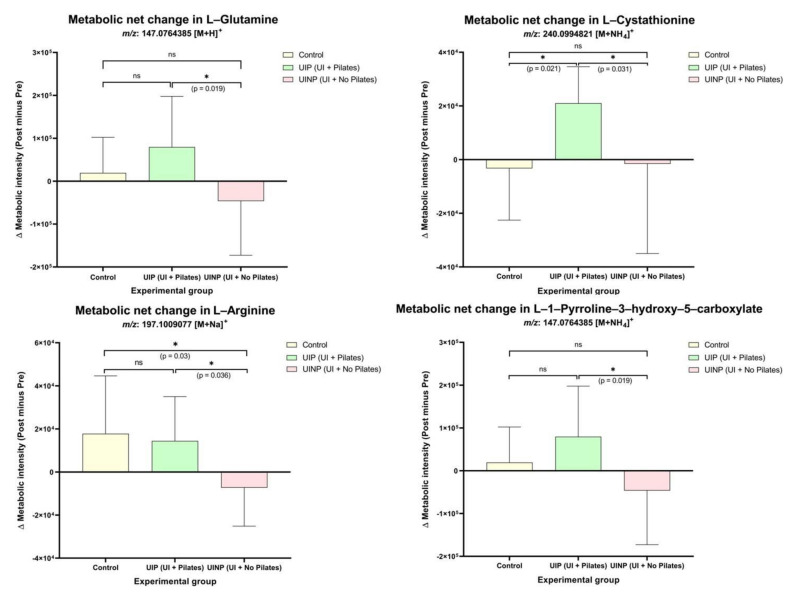
Net change in identified metabolic features associated with FMP ratio between pre- and post-intervention in Control, UIP, and UINP. Control: control group; UIP: urinary incontinence group with Pilates; UINP: urinary incontinence group with no Pilates; *m*/*z*: mass-to-charge ratio in ions that quantifies molecules in chemical or biological mixture; Δ metabolic intensity (post minus pre): metabolic net difference where MPI in pre-intervention substracted from post-intervention. * (*p* < 0.05): significantly different between groups. ns means non-significant.

In D-glutamine and D-glutamate metabolism, L-glutamine (*m*/*z*: 147.07 [M + H]^+^) in UIP showed significantly elevated metabolic intensity (*p* = 0.03) by 13.4% following 8-weeks intervention, whereas there were no significant ionic intensity changes in Control (3.1% ↑) and UINP (7.4% ↓) (*p* > 0.05) (Figure 4). UIP also showed a significantly up-regulated metabolome change (22.6% ↑, *p* = 0.0001) in L-cystathionine (*m*/*z*: 240.09 [M + NH_4_]^+^) under the glycine, serine, and threonine metabolism, however, attenuated intensity of L-cystathionine was found in both Control (2.7% ↓) and UINP (1.4% ↓) (*p* > 0.05). In arginine and proline metabolism, two metabolic features were also significantly increased in UIP (*p* < 0.05); L-arginine (*m*/*z*: 197.1 [M + Na]^+^) and L-1-pyrroline-3-hydroxy-5-carboxylate (*m*/*z*: 147.07 [M + NH_4_]^+^) were significantly up-regulated by 18.6% (*p* = 0.02) and 13.4% (*p* = 0.03), respectively. However, in Control and UINP, none of two metabolites in arginine and proline metabolism was significantly changed after 8-weeks (*p* > 0.05) (Figure 4). Additionally, to compare the metabolic net change between the three groups in each metabolite, net difference (post minus pre) of MPI between pre- and post-intervention was calculated, which was analyzed using one-way ANOVA. As shown in Figure 5, UIP showed a positive net change in all metabolic features, the calculated value of which was significantly higher compared to that of UINP (*p* < 0.05); the net metabolic intensity in UINP had a negative trend in all metabolites. Statistical significant net difference was not found between Control and UIP (*p* > 0.05), except for L-cystathionine (*p* = 0.021). In UIP, net change of L-cystathionine (*m*/*z*: 240.09 [M + NH4]+) was the most prominent (22.6% ↑), which was followed in numerical order by L-arginine (18.6% ↑), L-glutamine (13.4% ↑), and L-1-pyrroline-3-hydroxy-5-carboxylate (13.4% ↑). In UINP, the highest negative net change was found in L-arginine (*m*/*z*: 197.1 [M + Na]+) (8.6% ↓); Δ metabolic intensity in UIP (Δ14,453.0) was 200.8% significantly higher (*p* = 0.036) than that of UINP (Δ−7270.0) (metabolic net change in L-arginine, Figure 5). Comparing UIP and UINP, L-glutamine in UIP was up-regulated by 223.1% higher than UINP (UIP: Δ79,572.5; UINP: Δ−46,306.0) (*p* = 0.019) and not significantly differentiated from the Control (Δ19,286.3) (*p* > 0.05).

### 2.6. Change of Anthropometrical Data and FMP Ratio between Pre- and Post-Intervention

**Figure 6:**
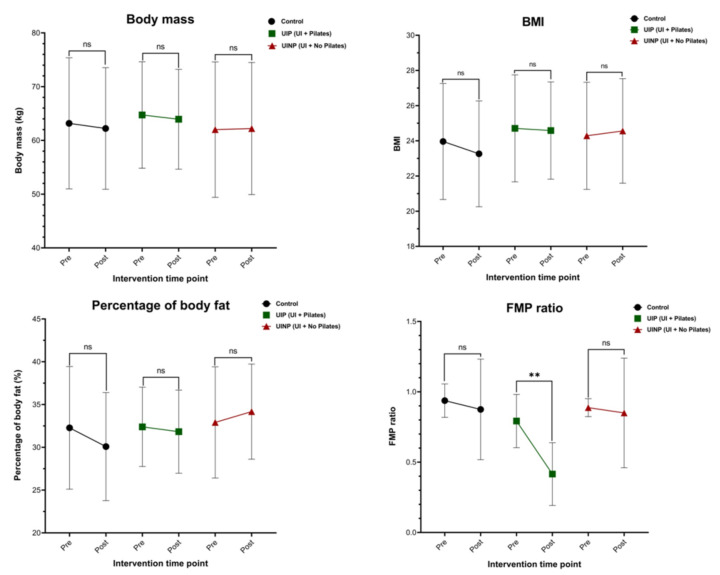
Change of anthropometrical data and FMP ratio between Pre- and Post-intervention in Control, UIP, and UINP. Control: control group; UIP: urinary incontinence group with Pilates; UINP: urinary incontinence group with no Pilates; FMP: functional movement of PFM; FMP ratio: calculated ratio where the movement distance of bladder base during PFM contraction state is divided by that during PFM resting state, contraction/resting state; pre: before 8-weeks intervention; post: after 8-weeks intervention. ** (*p* < 0.001): Significantly different from Pre. ns means non-significant.

After 8-weeks intervention, no significant changes in body mass, BMI, and percentage of body fat were found (*p* > 0.05) in all groups. However, UIP showed significantly decreased FMP ratio (*p* = 0.0002) by 48.1% (Pre: 0.79 ± 0.17; Post: 0.41 ± 0.21), but not in Control (*p* = 0.63, 7.4% ↓) (Pre: 0.95 ± 0.09; Post: 0.88 ± 0.38) and UINP (*p* = 0.61, 8.7% ↓) (Pre: 0.92 ± 0.07; Post: 0.84 ± 0.4) (Figure 6). Based on the interpretation of FMP ratio mentioned in Materials and Methods (4.4. Ultrasonography—Functional Movement of PFM), UIP indicating the smallest FMP ratio (0.41 ± 0.21) in post-intervention showed a significant functional improvement in PFM contractility following 8-weeks Oov Pilates program.

## 3. Discussion

The primary aim of this study was to provide the metabolomics perspectives whether the 8-weeks Pilates can induce the change of metabolic profiles in Korean women experiencing the symptoms of UI. To the best of our knowledge, this is first scientific approach that used the high resolution untargeted metabolomics to discover the underlying mechanism between Pilates and functional change of PFM known to be associated with UI symptoms. Our main findings demonstrated that, in UIP, Oov Pilates program can facilitate the functional movement of intrinsic muscles around pelvic floor area (Figure 6) as well as up-regulation of metabolic pathways and features (Table 2 and Figure 4). We also found that 8-weeks Oov Pilates was the physical intervention that can significantly maintain the intensity of metabolic feature, especially such as L-cystathionine (*m*/*z*: 240.09 [M + NH_4_]^+^), which was apparently opposite metabolomics trend in UINP (Figure 5). However, 8-weeks Pilates program using the Oov could not change physical activity-related anthropometrical factors such as body mass, BMI, and body fat percentage (Table 1 and Figure 6).

In PLS-DA (Figure 1), although the distinct metabolic discrimination between pre- and post-intervention was observed in all groups (Figure 1C–E), xMWAS [29] enabled the detection of candidate metabolic profiles that were biologically networked with anthropometrical data and FMP ratio, which was a key-analytical method that helped to select the essential metabolites interrelated with functional or clinical alteration of PFM.

As studies reported that Pilates has a clinically effective role in improving the strength and functional action of PFM in non-pregnant (Culligan et al., 2010) and nulliparous women [12] without UI symptoms, application of Pilates to UI individuals may also be a beneficial exercise intervention considering UI is associated with declined strength [13] and attenuated morphological change [5] in PFM. In our study, 8-weeks Oov Pilates program was a kinesiologically efficacious activity in ameliorating the functional action of PFM (Table 1 and Figure 6), which is consistent with the previous studies employing the Pilates exercise programs [10,11,12].

Although studies already demonstrated the efficacy of Pilate by measuring the PFM function per se in terms of managing UI symptoms [8,10,11,12,13], microscopic viewpoint regarding the underlying exercise mechanism of Pilates have not been extensively attempted, reported, and understood. In this context, our study raised a scientific speculation that Pilates generating the action of PFMT will affect the change of metabolic profiles in individuals with UI symptoms. As our result showed that FMP ratio was significantly decreased (*p* < 0.001) by 48.1% in only UIP (Figure 6), Pilates can be a beneficial method to effectively mobilize the PFM that helps maintain the continence of urination by assisting the pelvic organs [30]. Consequently, it is assumed that Pilates using Oov is an exercise method to enhance the PFM contraction, thereby robustly supporting the overall bladder base area and consistently maintaining the continence action in urethral passage for urinary functions [31]. More importantly, only in UIP, significant metabolic up-regulation was found in all identified metabolites, L-glutamine (*m*/*z*: 147.07 [M + H]^+^), L-cystathionine (*m*/*z*: 240.09 [M + NH_4_]^+^), L-arginine (*m*/*z*: 197.1 [M + Na]^+^), and L-1-pyrroline-3-hydroxy-5-carboxylate (*m*/*z*: 147.07 [M + NH_4_]^+^) (Figure 4), which assumes that decreased FMP ratio defined as the improvement of PFM function can be associated with enhanced metabolic features (Figure 4 and Figure 6).

Glutamine is known to be the most plentiful free amino acid in human muscle and plasma [32] and released from skeletal muscle considered as a primary tissue for glutamine synthesis to the circulation at 50 mmol/h [33]. In this sense, it is assumed that significant elevation of L-Glutamine (*m*/*z*: 147.07 [M + H]^+^) in UIP (Figure 4) may stand for increased formation of skeletal muscle fibers in PFM area, given that intramuscular concentration of glutamine is associated with the rate of protein synthesis [34,35] Furthermore, in UIP, there was also significantly increased metabolic intensity in L-arginine (*m*/*z*: 197.1 [M + Na]^+^) (*p* = 0.02, 18.6% ↑) (Figure 4) following 8-weeks intervention; its increment can be explained by the extent of L-glutamine change (*m*/*z*: 147.07 [M + H]^+^) (13.4% ↑) as glutamine is a major precursor for arginine in human plasma [36]. Studies of exercise science reported that short-term exercise induces the accumulation of plasma glutamine [37,38], whereas long-term physical activities accompanying strenuous physiological responses such as long distance running tends to reduce the glutamine concentration in blood [39,40,41], and in case by <500 μmol/L [37]. Scientific evidence for VO_2_max of Pilates has still remained inconclusive [42,43,44], however, Pilates may not be included in the category of exhaustive activities in terms of scientific common sense. Hence, provided that exercise intensity of Pilates meets the moderate and short-term level, significant increase of L-glutamine (*m*/*z*: 147.07 [M + H]^+^) in UIP is demonstrated by the view of metabolomics; Pilate is the activity that drives D-glutamine and D-glutamate metabolism as shown in pathway analysis (*p* = 0.0004, PI = 1.0) (Table 2 and Figure 3). Furthermore, since arginine can be originated from glutamine in terms of metabolic manner [45], hypothetically, significant elevation of L-glutamine (*m*/*z*: 147.07 [M + H]^+^) (*p* = 0.03, 13.4% ↑) in UIP may also affect the plasma concentration of L-arginine (*m*/*z*: 197.1 [M + Na]^+^) (*p* = 0.02, 18.6% ↑) if Pilates as a moderate and short-term exercise was properly performed. In human plasma under the fed state, it is reported that L-arginine concentration was approximately 200 μmol/L, and has been known to play a critical role in synthesizing the proline, glutamate, and creatine responsible for maintaining the cellular physiology [46] as well as serving as a precursor for the global protein synthesis in human body [47]. Based on the aforementioned scientific investigations, elevated blood concentration of either L-glutamine or L-arginine following 8-week Pilates program may be partly related to protein synthesis in PFM group, which can metabolically mediate the FMP and UI symptoms. However, our study did not measure the change of protein synthesis using proteomics or Western-blot so that the change of metabolic features (L-glutamine and L-arginine in Figure 4 and Figure 5) may not fully explain the morphological change of muscle fibers and remain speculative.

L-Cystathionine is formulated by transsulfuration of cystathionine β-synthase (CBS) through condensing L-homocysteine with L-serine [48], and has not been spotlighted whether its concentration or synthesis is affected by physical activities or sports. Although L-homocysteine functioning as adjacent precursor of L-cystathionine in an irreversible reaction was not identified in our study, a significant elevation of L-cystathionine (*m*/*z*: 240.09 [M + NH_4_]^+^) (*p* = 0.0001, 22.6% ↑) in UIP (Figure 4) may be a considerable metabolic candidate since L-cystathionine has been recognized to eliminate the production of superoxide radical [49,50], apoptosis [51], and endoplasmic reticulum stres [52]. In particular, studies found that L-cystathionine plays a crucial role in preventing the mitochondrial human apoptosis by excessive superoxide production [53] and mitochondria-dependent human apoptosis in vascular endothelial cell [54]. Therefore, increased intensity (Figure 4) and maintained net metabolic net change (Figure 5) in L-cystathionine (*m*/*z*: 240.09 [M + NH_4_]^+^) after 8-weeks Pilates program can be meaningful result when considering L-cystathionine can protect the biological property from oxidative stress and cellular apoptosis, which indicates that human vascular endothelial cell apoptosis may be delayed so that healthy condition of human vascular cells can be teleologically maintained in PFM groups. Although no studies have reported which type of physical activities or sports induce the change of L-cystathionine or cystathionine, our result indicated that 8-weeks Pilates program can be at least a tentative exercise intervention to amplify the metabolic intensity of L-Cystathionine (*m*/*z*: 240.09 [M + NH_4_]^+^). Our study found that UIP showed increased metabolic profiles in L-1-pyrroline-3-hydroxy-5-carboxylate (*m*/*z*: 147.07 [M + NH_4_]^+^) after 8-weeks Pilates program (Figure 4), however, there were no studies on how other physical activities or UI symptoms have an influence on the change of L-1-pyrroline-3-hydroxy-5-carboxylate. Although the human metabolomics database reported that the L-1-pyrroline-3-hydroxy-5-carboxylate belongs to organic compounds known as α-amino acids and moderately basic compound [55], studies found neither the functional implication between L-1-pyrroline-3-hydroxy-5-carboxylate and exercise intervention nor tangible action of L-1-pyrroline-3-hydroxy-5-carboxylate in human-derived samples.

In the clinical point of view, we expected that UINP under effect of UI symptoms will have a significantly differentiated metabolites that can be useful information to understand the symptoms. Our result showed, on the other hand, that four identified metabolites were not significantly changed following 8-weeks intervention although all metabolites in UINP showed a diminished tendency in metabolic intensity (Figure 4). Instead, in comparison of metabolic net change between UIP and UINP (Figure 5), occurrence of consistent negative metabolic net change in UINP may be a referential clinical indicator to characterize the UI symptoms in female population. Based on this metabolic phenomenon, it is speculated that, in UIP, maintained net metabolic status following 8-weeks Pilates-intervention could partly invigorate the physiological functions of PFM groups, which plays a pivotal role in ameliorating the FMP (Figure 5). Studies also reported candidate metabolic profiles related to lower urinary tract symptoms (LUTS) and stress urinary incontinence (SUI) [56,57], however, there were not UI-causing metabolic features in common between our result and two studies mentioned above.

In the realm of sports science, majority of studies focused on the change of exercise performance and anthropometric change following the oral supplementation of glutamine and arginine [58,59]. Therefore, as a benefit in return, how the exercise types, intensities, or duration determine the waxing and waning of metabolic profiles needs to be verified as Oov Pilates program in our study led to the change of specific metabolites associated with short-term and moderate exercise intensity.

In summary, 8-weeks Pilates program using Oov was the exercise intervention that can significantly improve FMP (48.1% ↑, *p* = 0.0002) and enhance the characteristics of human metabolomes, especially L-glutamine (*m*/*z*: 147.07 [M + H]^+^), L-cystathionine (*m*/*z*: 240.09 [M + NH_4_]^+^), and L-arginine (*m*/*z*: 197.1 [M + Na]^+^). The efficacy of 8-weeks Oov Pilates was also demonstrated in the view of metabolic net change when comparing the net change of UIP with that of UINP, especially in L-cystathionine (*m*/*z*: 240.09 [M + NH_4_]^+^) (Figure 5). These results highlighted that Oov Pilates can ameliorate the function of intrinsic deep muscles, PFM, (Figure 6) associated with clinically managing symptoms of UI, which was potentially related to up-regulated intensity of metabolic features as a positive physiological effect (Figure 4). Therefore, we speculate that 8-weeks Oov Pilates is an intervention that can achieve the improved functional movement of PFM and enhancement of metabolic feature, thereby ultimately mitigating the symptoms of UI in Korean women.

## 4. Materials and Methods

### 4.1. Ethical Approval

This study was approved by the Institutional Review Board (IRB) of Korea University and conducted in accordance with the ethical guidelines outlined by Korea University’s IRB (KUIRB-2019-0087-01). Written informed consent was obtained from all participants before participation of the study.

### 4.2. Participants

A total of twenty-nine subjects (*n* = 29) (Age: 43.5 ± 6.5 years; Height: 159.9 ± 7.7 cm; Body mass: 63.2 ± 11.1 kg; Body mass index, BMI: 24.4 ± 3.0) were recruited from Sejong City in South Korea, and reported the pre-menopausal state with regular menstruation. Twenty-one subjects (*n* = 21) experienced the symptoms of UI more than one and less than ten times within a week, and were randomly assigned into two groups, UI group with Pilates (UIP, *n* = 13) (Age: 42.4 ± 5.0 years; Height: 160.7 ± 5.1 cm; Body mass: 64.1 ± 10.3 kg; Body mass index, BMI: 24.7 ± 3.0) and UI group with no Pilates (UINP, *n* = 8) (Age: 46.4 ± 8.5 years; Height: 156.6 ± 10.5 cm; Body mass: 62.0 ± 12.6 kg; Body mass index, BMI: 24.3 ± 3.0) (Table 1). Control group (Control, *n* = 8) (Age: 42.5 ± 6.2 years; Height: 161.9 ± 8.3 cm; Body mass: 63.2 ± 12.2 kg; Body mass index, BMI: 24.0 ± 3.3) had no clinical symptoms of UI. UIP and UINP had urological and gynecological-related symptoms, however, underwent neither the invasive surgical treatments nor non-invasive interventions including hormonal oral-medication. All subjects were instructed to maintain their normal activities during their participation, but to refrain from caffeine, alcohol, and strenuous physical activities on the day of blood collection and ultrasonography test.

### 4.3. Exercise Intervention—Pilates

UIP performed the Pilates using the Oov known as an ergonomic-shape durable form that helps to achieve the natural curve of the spine and motor learning by mimicking the anatomical movement of spine [60]. UIP conducted 60-min Oov Pilates 3 times weekly for 8 weeks, which was composed of 10-min warm-up, 40-min main exercise, and 10-min cool-down and designed to mainly enhance the abdominal muscles and PFM. To maintain the exercise intensity in main exercise, target heart rate (THR) suggested by Karvonen [61] and rating of perceived exertion (RPE) were individually measured during 8 weeks so that each participant could decide their own intensity for upcoming sessions. Until by 4-weeks of main exercise, THR was decided by 55–75% of heart rate reserve (HRR), and RPE of 11–13 was administrated to the participants. Exercise intensity for main exercise between 5 and 8-weeks period was progressively increased to 65–75% of HRR and RPE of 13–15.

### 4.4. Ultrasonography—Functional Movement of PFM

Functional movement of PFM (FMP) was measured by SONON Convex 300C (Healcerion, Seoul, Korea) equipped with multiple frequencies technology (5.0, 7.5, 10.0 MHz) and wireless convex array transducer. To achieve the accurate data collection, we followed previously approved clinical procedures for the measurement [13,62,63], and one designated professional practitioner operated the ultrasonography apparatus to observe the FMP. For the acquisition of vivid ultrasonography data in bladder base, subjects were asked not to visit the restroom for the urination 1 h before the examination, and to be hydrated by drinking 2 cups of water in 30 min before the examination. During the assessment, the angle of knee was 60° in supine position; block-shaped probe was located above pubic bone at the sagittal angle and steered to measure toward the direction of transverse plane by the angle of 15–30°. FMP was equivalently created by contracting the muscle groups of anus. After practicing the contraction of anus area once, baseline FMP was measured during the resting period, and maximal contraction of anus was maintained for 3 s in order to measure the maximal FMP [64]. Maximal FMP was measured 3 times with 1 min resting period between sessions [65], and averaged FMP was used for final data analysis. FMP ratio was the calculated value where the movement distance of bladder base during PFM’s contraction state is divided by that during PFM’s resting state; small ratio represents the ameliorated or improved FMP, and vice versa.

### 4.5. Hematology and Blood Sample Preparation

For all subjects, blood sample collection was performed at the time point of pre and post-Pilates intervention. Before the experimental day, all subjects were asked to follow ≥8 h overnight fast and fluid restriction, non-caffeinated drinks, and non-intense physical activity. When arrived in laboratory next day, they were asked to maintain 10 min-controlled sitting posture to prevent the plasma volume change and variation [66]. Whole blood was collected from prominent forearm antecubital vein by 4.0 mL K_2_ EDTA (Lavender-top) Vacutainer method. The tube was immediately centrifuged by 1600× *g* for 15 min, and plasma sample as a supernatant was obtained and transferred to Microtainer tube by 500 µL aliquot that was stored in cryogenic freezer (at −80 °C) until use. Total of 50 µL aliquot of plasma was added to the mixture of 100 µL containing 95 µL of LC-MS grade acetonitrile (ACN) and 5 µL of three isotope standards ([3-Methyl-^13^C]-Caffeine; [Dimethyl-D6]-N,N-Diethyl-M-Toluamide; [^13^C5, ^15^N]-L-Methionine) (1:2, *v*/*v*). The samples were vortexed for 1 min and consecutively centrifuged at 13,000 rpm at 4 °C for 10 min to perform the protein precipitation and extraction of metabolic feature. The supernatant containing the polar metabolic substances was obtained and consecutively transferred to the LC-MS vial for LC-MS/MS measurement.

### 4.6. Untargeted Data Acquisition Using LC-MS Q-TOF

An high resolution untargeted metabolomics was used to acquire the metabolic profiles of plasma samples [67]. The metabolomics data acquisition was performed through high performance liquid chromatography system (HPLC) (1290 Infinity, Agilent, City of Santa Clara, CA, USA) equipped with Agilent 6550 iFunnel quadruple-time of flight liquid chromatography/mass spectrometry (Q-TOF LC/MS) (Agilent, CA, USA). The separation in LC was achieved by Hypersil GOLD aQ C_18_ column (100 mm × 2.1 mm; particle size of 1.9 μm) (Thermo, Waltham, MA, USA) of which system temperature was maintained at 45 °C. For the mobile phases, solvent A was LC-MS grade water (H_2_O) (JT-Baker, USA) with 0.1% formic acid (CH_2_O_2_) (Sigma-Aldrich, St. Louis, MO, USA), and solvent B was the LC-MS grade acetonitrile (CH_3_CN) (ACN) (J.T.Baker, Avantor, Allentown, PA, USA) mixed with 0.1% formic acid. The HPLC mixing gradient of aforementioned mobile phases was programmed as follow: 0.0–1.0 min, 5% in solvent B; 1.0–9.0 min, 45% solvent B; 9.0–12.0 min, 90% solvent B; 12.0–13.5 min, 90% solvent B; 13.5–13.6 min, 5% solvent B, which was conducted for 15 min in total. The treated plasma sample injection volume by auto-sampler and solvent flow rate were 3 μL and 0.4 mL/min, respectively. The capillary voltage was set at 3.5 kV, and temperature of the drying and sheath gases were 250 °C. To detect the mass/charge ratio (*m*/*z*) in ions, selection criteria was set at between the *m*/*z* of 50.0 and 1000.0 with resolution of 20,000, which was obtained through positive electro-spray ionization (+ESI) mode. All plasma samples in LC-MS were randomly run in triplicate to guarantee the potential statistical reliability and reproducibility. An equivalent volume (150 µL) of 100% ACN was added to the actual samples’ line-up to ensure the cleaning of sample injection needle and prevent the sample contamination. Since randomly assigned 29 plasma samples were ran during the individual day, and may have potential non-biological factors during the metabolomics measurement, we performed a batch effect correction by using R-based xMSanalyzer designed to conduct the automatic correction on the peak data through the improved metabolic peak detection [68].

The characteristics of metabolites was specified by *m*/*z*, retention time (R/T), and intensity [69], and real-time spectra using total ion chromatogram (TIC) and base peak chromatogram (BPC) were also observed and check during the measurement.

### 4.7. LC/MS Data Processing and Statistical Analysis

#### 4.7.1. Data Extraction of LC/MS Raw Files

The spectral LC-MS raw data (‘.d’ type file) was converted to ‘.MZXML’ file by using MSConvert (Proteowizard, http://proteowizard.sourceforge.net/index.html (accessed on 16 July 2020), command-line tool for mass spectrometry file conversion. Then, the converted files (MZXML) were processed through apLCMS (R-driven package, ver. 3.4.3) that provides computational algorithm to achieve the metabolic feature detection and quantification as well as accurate feature alignment [70], which included *m*/*z*, R/T, and metabolic peak intensity (MPI). The output files of apLCMS were evaluated and corrected by xMSanalyzer that enables the integration of apLCMS files, evaluation of sample quality, metabolic feature consistency, and batch-effect correction in triplicate measured plasma samples [68].

#### 4.7.2. Discriminative Analysis between Pre and Post-Intervention in Control, UIP, and UINP

To verify discrimination of metabolic profiles in and between pre- and post-intervention in Control, UIP, and UINP (Figure 1), median-summarized 6574 metabolic features containing *m*/*z*, RT, and MPI acquired by xMSanalyzer were entered to SIMCA 14.1 (Umetrics AB, Umea, Sweden) with unit variance (UV) scaling function in order to produce the partial least squares-discriminant analysis (PLS-DA) known as supervised multivariate analysis. In addition, the analytical procedure of 7-fold cross validation embedded in SIMCA 14.1 by default could evaluate the quality of PLS-DA model and minimize the risk of model’s over-fitting. To confirm the goodness of fit and predictive ability in PLS-DA model, both R^2^ and Q^2^ were assessed in three experimental groups.

#### 4.7.3. Integrative and Differential Network Analysis Using xMWAS

The median-summarized data including *m*/*z*, R/T, and MPI was obtained by xMSanalyzer, and used to operate the xMWAS (v0.552, https://kuppal.shinyapps.io/xmwas (accessed on 10 September 2020) that facilitates the data integration, differential network analysis using topological approach, and visualized factor clustering [29] between metabolic profiles and comparative data (Y1: Body mass; Y2: BMI; Y3: Body fat percentage; and Y4: FMP ratio) (Figure 2). When visualizing the clusters between metabolic profiles and fours variables (Y1, Y2, Y3, and Y4), all of each raw dataset were entered and analyzed in xMWAS at once. In Control, UIP, and UINP, to confirm the correlation between metabolomic profiles and Y4, cluster information table obtained from xMWAS was systemically filtered based on Y4, which consequently displayed the key metabolites associated with FMP ratio defined as a functional change of PFM.

#### 4.7.4. Metabolomics Analysis for Metabolic Pathway

To identify the metabolic pathways and potential metabolism affected by 8-weeks intervention in Control, UIP, and UINP (Table 2 and Figure 3), dataset including *m*/*z* and R/T from xMWAS (v0.552, https://kuppal.shinyapps.io/xmwas (accessed on 10 September 2020) was annotated using Human Metabolome Database (HMDB) (https://hmdb.ca (accessed on 15 November 2020) [71] known as web-enabled metabolic database providing *m*/*z*, KEGG identifier, and compound name; we used *m*/*z* error tolerance of ± 10 ppm (± 0.001%) to minimize the inclusion of irrelevantly annotated metabolites. For pathway analysis, KEGG ID (https://www.genome.jp/kegg/tool/conv_id.html (accessed on 18 November 2020) produced by HMDB was entered into MetaboAnalyst 4.0 (https://www.metaboanalyst.ca (accessed on 20 November 2020), web-based comprehensive metabolomic analysis [72], in order to produce the overview of pathway analysis in metabolites (Table 2). When deciding the potential metabolic pathways in MetaboAnalyst 4.0, the Summary of Metabolome View and Table of Detected Pathways were equivalently considered in selecting putative metabolites in Control, UIP, and UINP.

### 4.8. Statistical Analysis and Analytical Data Visualization

To visualize the analytical data, MPI of selected metabolites was analyzed and illustrated using the GraphPad Prism software Ver. 8.0 (GraphPad, San Diego, CA, USA), which was performed between pre- and post-intervention. Data were presented as mean ± SD, and all statistical significance level was set at *p* < 0.05.

## Figures and Tables

**Table 1:** Demographic characteristics and pelvic floor muscle (PFM) function of participants.

Group	Control		UIP (UI + Pilates)		UINP (UI + No Pilates)	
Participant (*n*)	8		13		8	
Age (yr)	42.5 ± 6.2		42.4 ± 5.1		46.4 ± 8.5	
Height (cm)	161.9 ± 8.3		160.7 ± 5.1		156.6 ± 10.5	
	Pre	Post	Pre	Post	Pre	Post
Body mass (kg)	63.2 ± 12.2	62.2 ± 11.3	64.1 ± 10.2	63.8 ± 9.5	62.0 ± 12.6	62.2 ± 12.3
BMI	23.9 ± 3.3	23.3 ± 3.0	24.7 ± 3.0	24.6 ± 2.8	24.3 ± 3.1	24.6 ± 2.9
Body fat (%)	32.3 ± 7.2	30.1 ± 6.3	32.4 ± 4.6	31.8 ± 4.6	32.9 ± 6.5	34.2 ± 5.6
FMP ratio	0.9 ± 0.1	0.9 ± 0.4	0.8 ± 0.2	0.4 ± 0.2 *	0.9 ± 0.1	0.8 ± 0.4

PFM: Pelvic floor muscle; Control: control group; UIP: urinary incontinence group with Pilates; UINP: urinary incontinence group with no Pilates; Pre: measurement conducted before 8-weeks intervention; Post: measurement conducted after 8-weeks intervention; BMI: body mass index; FMP: functional movement of PFM; FMP ratio: calculated ratio where the movement distance of bladder base during PFM contraction state is divided by that during PFM resting state, contraction/resting state; * (*p* < 0.05): Significantly different from Pre. Values are expressed as mean ± SD.

**Table 2:** Statistical overview of metabolic pathways associated with FMP ratio.

Group	Pathway Name (Metabolism)	Match Status	*p*-Value	−Log(*p*)	Pathway Impact
**Control**	D-Glutamine and D-glutamate	2/6	0.2845	1.2571	0
	Glycine, serine and threonine	4/33	0.8577	0.1535	0.0029
	Arginine and proline	3/38	0.9745	0.0258	0.1106
**UIP**	D-Glutamine and D-glutamate	4/6	0.0004	7.7956	1.0
	Glycine, serine and threonine	8/33	0.0023	6.0662	0.3381
	Arginine and proline	8/38	0.0059	5.1278	0.2205
**UINP**	D-Glutamine and D-glutamate	N.I	N.I	N.I	N.I
	Glycine, serine and threonine	6/33	0.6212	0.4762	0.0503
	Arginine and proline	7/38	0.6053	0.5020	0.3554

Match status: Value indicating the number of matched metabolic feature in pathway, e.g., 2/6 is showing two matched metabolic feature of total six features in D-Glutamine and D-glutamate metabolism pathway; pathway impact: statistical value calculated based on the metabolic importance of matched metabolites in each pathway, which uses the pathway topology analysis using the relative-betweeness centrality. Control: control group; UIP: urinary incontinence group with Pilates; UINP: urinary incontinence group with no Pilates; N.I: not identified.

## Data Availability

The data presented in this study are available on request from the corresponding author. Data is available on request from the corresponding author because we are managing the raw data and results based on laboratory policy.
